# The Impact of Discharge Planning on Enhancing Independence in Ischaemic Stroke Patients: A Post-hospitalisation Rehabilitation Approach

**DOI:** 10.21315/mjms-01-2025-027

**Published:** 2025-04-30

**Authors:** Upik Rahmi, Lisna Anisa Fitriana, Suci Tuty Putri, Septian Andriyani, Farida Murtiani

**Affiliations:** 1 Faculty of Sport and Health Education, Universitas Pendidikan Indonesia, Bandung, Indonesia; 2 Clinical Research Unit, Sulianti Saroso Infectious Disease Hospital, Jakarta, Indonesia

**Keywords:** discharge planning, ischaemic strokes, activities of daily living, rehabilitation, disability evaluation

## Abstract

**Background:**

Ischaemic stroke is a leading cause of physical disability, significantly impacting patients’ independence in daily activities. The inability to perform basic activities of daily living poses a major challenge for post-stroke patients, affecting their quality of life. Discharge planning is a crucial approach in stroke rehabilitation aimed at enhancing patient independence after hospitalisation. This study evaluated the impact of structured discharge planning on the independence of ischaemic stroke patients.

**Methods:**

A quasi-experimental design was employed, with the intervention group receiving discharge planning and the control group receiving no intervention. Patient independence was assessed by applying the Barthel Index, evaluating their ability to perform daily activities before and after the intervention.

**Results:**

The intervention significantly improved the independence levels of the participants in the intervention group, as measured by the Barthel Index, with their mean score increasing from 2.8 to 11.3. In comparison, the control group also showed improvement, with the mean score rising from 5.7 to 10.1.

**Conclusion:**

Discharge planning effectively enhances the independence of patients with ischaemic stroke. Implementing this programme in hospitals is expected to reduce patient dependency and improve the quality of life.

## Introduction

Discharge planning is an important aspect of ensuring the readiness of stroke patients to undergo recovery at home and manage the challenges arising after a stroke (1). Proper discharge planning significantly improves patient readiness, as observed by readiness score improvement after the intervention (1). It not only focuses on the patient but also involves the family in this process. By educating families and ensuring their preparedness to support patients at home, discharge planning contributes to improved quality of life for stroke patients (2). In addition, a thorough evaluation of the patient’s health needs and changes at home for their support ensures a successful recovery process. Thus, a comprehensive and structured discharge plan can significantly improve the transition from hospital care to home care for stroke patients (2).

Previous studies report that proper discharge planning improves the quality of life of stroke patients through increased physical activity, the prevention of complications, and medication adherence (3). Involving families in the process and providing appropriate education to patients is associated with improved support and long-term outcomes. In addition, studies show that effective discharge planning reduces readmission, complications, and mortality rates while enhancing the quality of life, knowledge and satisfaction for stroke survivors and their caregivers (4). This strategy also promotes long-term adherence to treatment, which is an important factor for preventing stroke recurrence and improving overall health. Structured discharge planning significantly improves patient readiness for discharge, as supported by a study that reported a *P*-value of 0.001 in both intervention and control groups. The importance of a multidisciplinary approach to discharge planning is emphasised, ensuring collaboration between healthcare teams, patients, and families (5). Thus, effective discharge planning is a critical component in the rehabilitation process of stroke patients and contributes to improving the patient’s quality of life, well-being, and recovery.

Several studies in other countries also report that similar interventions in discharge planning can enhance stroke patients’ outcomes. For example, in Scandinavia, implementing educational programmes enhanced the understanding of stroke patient care, leading to reduced readmission rates. Another study in Australia reported that using patient decision aids during the discharge process improved communication about expected outcomes, thereby strengthening patient involvement in the care process. These findings suggest that integrated strategies across different countries can significantly improve the quality of care and accelerate the recovery path for stroke patients, as it is important to implement them broadly to avoid any negative long-term outcomes and delay the recovery process (6, 7). This study emphasises the effectiveness of the Patient-Focused Departure Programme for Caregivers, which serves to improve the clarity and completeness of discharge instructions. Caregivers who participated in the programme showed better ability and understanding of managing stroke care compared to those who received standard discharge planning. This approach prepares caregivers to ensure an effective transition from hospital to home, ultimately supporting optimal patient recovery (8).

Although various studies have shown that homecoming planning plays an important role in improving family readiness, reducing disability, and improving the survival of patients with ischaemic stroke, there is a research gap related to the independence of post-stroke patients. Most studies emphasised medication adherence and preventing recurrence but overlooked functional independence, a key determinant of long-term quality of life in stroke patients (1, 9). Patients’ independence in carrying out activities of daily living (ADLs), such as self-care, mobility, and social interaction, is still often ignored as a parameter for successful discharge planning. In addition, no discharge planning model individualises patient care based on before and after stroke independence or actively involves caregivers in supporting sustainable rehabilitation. The lack of in-depth research exploring the effectiveness of discharge planning to increase patient independence is an essential factor for further study. Therefore, the study’s objective is to develop and test a comprehensive and focused discharge planning model focused on improving the independence of patients after ischaemic stroke. This model enables functional assessment and helps tailor rehabilitation based on individual patient needs, as well as caregiver involvement, to support an effective transition from hospital to home, fostering the patient’s long-term independence and quality of life (10–13). The research question focused on how structured discharge planning influences the independence levels of these patients after hospital discharge.

## Methods

The current study involved 43 ischaemic stroke patients who were hospitalised. The inclusion criteria were as follows: patients who were discharged after hospitalisation, adult patients admitted to the hospital, patients who survived at least 10 days after discharge from the hospital, and patients living in the community before being treated. The exclusion criteria were patients with haemorrhagic stroke. The patients’ ages ranged from 26 years to 74 years. The discharge planning implementation was assessed after the nurse explained to the patient following the discharge planning format available at the hospital. This includes health services required for the discharge of patients, health education for patients and families, the needs of patients after discharge, signs and symptoms of disease recurrence, the drugs given (dosage, how to use, side effects), and foods that should be consumed and that should be avoided.

The discharge planning initiative started from one of the public hospitals in Indonesia, and patient independence is measured using the Barthel Index, which consists of 10 indicators, namely eating, bathing, self-care, dressing, urinating, defecating, toilet use, transfer, mobility, and going up and down stairs (14).

The research procedure was carried out by providing discharge planning to stroke patients who returned from hospitalisation. The discharge planning used included 18 question items. Ten days after the patient was discharged, the independence measurement was carried out using the Barthel Index questionnaire to evaluate changes in the level of independence during the period, and the Barthel Index was self-reported by researchers. Data were statistically analysed using the mean, median, and standard deviation values. Before the parametric statistical test was conducted, a data normality test using the Shapiro-Wilk test was applied. The level of patient independence was measured before and after the discharge planning intervention to assess the changes that occurred.

## Results

The majority of respondents belong to the age group over 55 years old. The majority of respondents were women (25 in number; 58.14%). More than half of the respondents, i.e., 22 people (51.16%), were housewives. Regarding health financing sources, most respondents were 26 people (60.47%). Regarding hospitalisation experience, most respondents, 30 people (69.77%), had never been hospitalised (Table 1).

Independence level before intervention based on the Barthel Index, the intervention group consisted of 20 respondents with a mean value of 2.8 and a confidence interval (95% CI) between 1.5 to 4.1. On the other hand, the control group, consisting of 23 respondents, had a higher average independence score of 5.7 and a confidence interval (95% CI) of 3.2 to 8.1. Post-implementation of the intervention based on the Barthel Index, the intervention group consisted of 20 people with a mean value of 11.3 and a confidence interval (95% CI) between 9.9 and 12.8. Meanwhile, the average Barthel Index was 10.1 in the control group with 23 respondents, and the confidence interval (95% CI) was 7.7 to 12.5 (Table 2).

**Figure 1:**
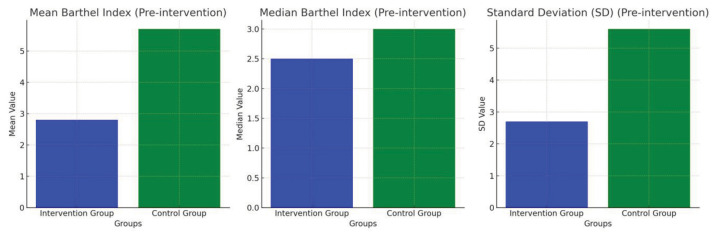
Ischaemic stroke independence rate before intervention

**Figure 2:**
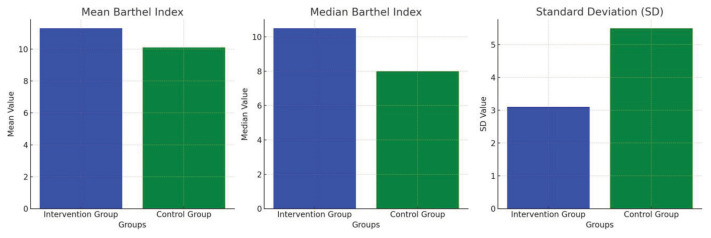
Independence rate of ischaemic stroke patients after intervention

An overview of the results is visualised as a graphical representation. Before the intervention, the control group showed a higher level of independence than the intervention group, which was reflected in the difference in the mean and median values between the two groups (Figure 1). After the intervention, the intervention group had a higher average level of independence than the control group, which indicates the potential positive effect of the intervention on increasing independence. In addition, the variability of values in the control group was greater, as indicated by the standard deviation and wider range of values, indicating a greater difference in the level of independence in this group (Figure 2).

## Discussion

The findings showed that the intervention succeeded in increasing the independence of patients with stroke. Patients who received the intervention had an improved ability to perform ADLs compared to the control group. Most patients in the intervention group achieved a better level of independence, which was reflected in a higher median outcome than the control group. In addition, the variation in outcomes across the intervention groups was smaller and more consistent, indicating that the intervention provided more equitable patient benefits. The intervention group also showed a narrower range of outcomes, signalling a more stable level of independence than the control group, with more varied results. Regarding the reliability of outcomes, a narrower confidence interval in the intervention group confirmed that the intervention had a significant and reliable positive effect on improving patient independence.

The findings of our research align with those of previous studies and show the efficiency of rehabilitation interventions in enhancing the independence of stroke patients. The study by Quinn et al. (14) emphasised that applying the Barthel Index to measure independence in stroke patients is a valid method to assess their functional ability to carry out ADLs. Other studies also report similar findings that structured discharge planning and rehabilitative interventions effectively improve patients’ motor skills and basic daily activities after stroke (15). The present study found that the right intervention can accentuate functional recovery and lead to better long-term outcomes. The lower variation and more consistent results in the intervention groups reflect the benefits of a focused and personalised approach to intervention, as reported earlier, to improve rehabilitation outcomes significantly (16). Therefore, these findings substantiate that timely and targeted rehabilitative interventions can significantly improve the independence of stroke patients.

In addition, the higher median values in the intervention group showed that most patients in this group achieved better independence than those in the control group. This finding is in line with research by Langhorne et al. (15), who found that intensive rehabilitation interventions significantly accelerated functional recovery in stroke patients. The smaller standard deviation in the intervention group indicates more consistent results with lower outcome variability, suggesting evenly distributed benefits. The narrower range of values in the intervention group also showed that the variation in the patient’s level of independence was more controlled than in the control group, where the results were more varied. A narrower confidence interval in the intervention group strengthened the reliability of these findings, supporting the effectiveness of the interventions. These results support the previous findings (16), highlighting the significance of a structured and systematic intervention approach for better stroke rehabilitation outcomes.

However, unexpected results were observed, such as a significant increase in independence among the control group patients despite the lack of intervention. This may be due to external factors, such as family support, the patient’s initiative, or access to additional rehabilitation that was not recorded in the study. Quinn et al. (14) suggested that individual motivation and social support may significantly impact stroke recovery, explaining the variation observed in the control group.

Research has been conducted on the effect of discharge planning on independence in ischaemic stroke patients, which is comprehensive in significantly increasing the independence of ischaemic stroke patients, especially in the aspects of ADLs and mobility (17). Another study indicates that these planning interventions significantly reduce disability and enhance patients’ performance in daily activities, quality of life, and overall satisfaction. Thus, effective discharge planning not only impacts the physical independence of patients but also improves their psychological aspects and quality of life (18). Post-implementation of the programme, ischaemic stroke patients showed mild dependence, while previously, two-thirds of them were in a highly dependent condition (19). These results underscore the importance of a structured approach to the programme before patients are discharged, specifically for patients having severe limitations immediately after hospital admission.

Although hospital recovery is important, the findings indicate that post-discharge recovery can occur independently of hospital recovery status. This shows that ischaemic stroke patients develop independence at home, particularly with adequate family and medical support through discharge planning (20). Family-based interventions and physical activities are important components of discharge planning. Studies have shown that family empowerment and exercise-based strategies significantly improve ischaemic stroke patients’ self-care independence and mobility (21). Families who play an active role in patient rehabilitation can provide positive encouragement and increase patients’ confidence in carrying out daily activities. In addition, regular physical exercise helps improve the patient’s muscle strength, flexibility, and motor ability, which directly contributes to increased independence (20).

Mobility independence is also an important indicator in discharge planning. After discharge from the hospital, approximately 55% of ischaemic stroke patients who were previously unable to walk achieved a level of partial or full mobility autonomy, including the ability to climb stairs and walk inside and outside the home. This progress indicated that the right intervention can positively impact patients with significant mobility limitations, and they can achieve better independence after discharge from the hospital (22). An initial assessment of post-stroke ADLs can also predict a patient’s future level of independence. Early dependence within the first two days often indicates a higher likelihood of dependence at 3–12 months after (23). Therefore, good and structured discharge planning is an important component of the care of patients with ischaemic stroke. This process requires multidisciplinary collaboration integrating diverse ontological, epistemological, and axiological approaches for effective post-discharge care (24). A structured discharge plan enhances patient independence by improving physiological function, cognitive understanding, and self-efficacy while reducing stress and family care burden (25). Research in a private hospital in Indonesia also showed that discharge planning significantly improved self-care behaviour in patients with ischaemic stroke. In contrast, there were no significant changes in the other hospitals after the implementation of every programme. This suggests that the effectiveness of discharge planning can vary depending on the approach and resources available at each health facility (26).

Overall, discharge planning plays a significant role in increasing the independence of ischaemic stroke patients. With the right intervention, more than half of stroke patients who had previously experienced heavy dependence showed a significant improvement in their level of independence after discharge from the hospital (27, 28).

The current study also demonstrated that family empowerment-based planning is essential. These are ongoing educational interventions directed towards caregivers and families as a means for them to understand that they can participate in the patient rehabilitation process. (20). With adequate knowledge about medications, nutrition, and rehabilitation needs, one can ensure faster recovery and promote home care quality (22). Through proper support, ischaemic stroke patients can develop independence in mobility and daily self-care. Comprehensive discharge planning should also incorporate formal teaching regarding the process of disease management, treatment, and rehabilitation based on the particular needs of the patient. Applying learning algorithms to identify patients’ needs can also help predict treatment outcomes, thereby increasing their independence after discharge from the hospital (29). The Early Supported Discharge (ESD) programme is an example of successful discharge planning implementation. By providing home-based rehabilitation, the ESD programme provides aid for better patient functional outcomes and reduces hospital stay length. This justifies effective discharge planning being influential in the quality of life of ischaemic stroke patients (30).

Limitations persist, although this study emphasises the crucial process of discharge planning to improve the independence of ischaemic stroke patients. First, it focused on the level of independence of post-stroke patients after receiving discharge planning interventions before returning home or to the community. Although the results were positive, other factors, such as social and environmental conditions, also affect recovery outcomes. Further, there was insufficient monitoring of the patient’s interactions with their surroundings. Second, there were no rigorous guidelines regarding the frequency or quality of home visits, and the study lacked structured monitoring. This raises the question of whether discharge planning or other unquantifiable factors contributed to the favourable results. Discharge planning must incorporate customised home visits to guarantee continued supervision and support, avoiding gaps in post-discharge care.

However, the main strength of this study is its emphasis on discharge planning to enhance functional recovery and prevent long-term disability. With proper education and planned support, ischaemic stroke patients can achieve greater independence in performing daily activities after discharge from the hospital. Future research is recommended with stricter home and tailored discharge planning based on personalised requirements. Thus, the effectiveness of discharge planning can be ascertained and directly associated with increased patient independence. Integrating comprehensive discharge planning in post-stroke care is highly recommended for better outcomes.

Long-term follow-up 10 days post-discharge was used to evaluate the progress of the patient’s mobility and ADLs. This assessment helps the care team determine the need for further intervention. Further consultation with a physical or occupational therapist can be reinforced with appropriate exercise to the patient’s needs, thereby improving strength and endurance. In addition, monitoring patients’ mental health is crucial to preventing depression or anxiety. Mental support programmes or support groups and ongoing health education equip caregivers with the necessary knowledge to support patients effectively.

## Conclusion

It was observed that discharge planning significantly influenced the level of independence of ischaemic stroke patients. Through structured and thorough discharge planning, patients achieve better mobility and their ability to carry out their ADLs. The study also indicated that patients who received a good discharge planning intervention showed a greater improvement in independence compared to the control group that did not receive a similar intervention.

## Figures and Tables

**Table 1:** Characteristics respondents stroke ischaemic (*n* = 43)

Characteristics of respondents	*n*	%
Age		
26–35 years old	2	4.65
36–45 years old	6	13.95
46–55 years old	15	34.88
> 55 years	20	46.51
Gender		0.00
Woman	25	58.14
Men	18	41.86
Work		0.00
Private sector/trader/farmer	5	11.63
Housewives	22	51.16
Honorary personnel	5	11.63
Pensioner	6	13.95
Day labourers	5	11.63
Source of financing		0.00
General/cash	7	16.28
Loans	6	13.95
Public health insurance	4	9.30
Health insurance	26	60.47
Hospital nursing experience		
Yes	13	30.23
No	30	69.77

**Table 2:** Independence level of ischaemic stroke patients before intervention and after intervention

Barthel Index	*n*	Mean	Median	SD	Min–Max	95% CI
Before (pretest)						
Intervention groups	20	2.8	2.5	2.7	0–9	1.5–4.1
Control group	23	5.7	3	5.6	0–17	3.2–8.1
After (postest)						
Intervention groups	20	11.3	10.5	3.1	8–19	9.9–12.8
Control group	23	10.1	8	5.5	3–20	7.7–12.5
